# Umbilical Cord Blood Natural Killer Cells, Their Characteristics, and Potential Clinical Applications

**DOI:** 10.3389/fimmu.2017.00329

**Published:** 2017-03-23

**Authors:** Anushruti Sarvaria, Dunia Jawdat, J. Alejandro Madrigal, Aurore Saudemont

**Affiliations:** ^1^Anthony Nolan Research Institute, London, UK; ^2^Cancer Institute, University College London, London, UK; ^3^King Abdullah International Medical Research Center, Riyadh, Saudi Arabia

**Keywords:** natural killer cells, umbilical cord blood, immunotherapy, cancer, hematopoietic stem cells

## Abstract

Natural killer (NK) cells are lymphocytes of the innate immune system able to kill different targets such as cancer cells and virally infected cells without prior activation making then attractive candidates for cancer immunotherapy. Umbilical cord blood (UCB) has become a source of hematopoietic stem cells for transplantation but as we gain a better understanding of the characteristics of each immune cell that UCB contains, we will also be able to develop new cell therapies for cancer. In this review, we present what is currently known of the phenotype and functions of UCB NK cells and how these cells could be used in the future for cancer immunotherapy.

## Introduction

Natural killer (NK) cells are lymphocytes of the innate immune system that exhibit cytotoxicity toward cancer cells and virus-infected cells and have the capacity to produce cytokines such as interferon-γ (IFN-γ) and tumor necrosis factor-α (TNF-α) in response to stimuli. NK cells are defined as CD56^+^CD3^−^ cells and can be divided into two main subsets according to their expression of CD56 and CD16. CD56^dim^CD16^+^ NK cells (CD56^dim^ NK cells) are cytotoxic NK cells capable to mediate direct killing of target cells *via* exocytosis of granules containing granzyme B and perforin, activation of cell death pathways such as TRAIL or FAS/FAS-L or *via* antibody-dependent cellular cytotoxicity. CD56^bright^CD16^−/low^ NK cells (CD56^bright^ NK cells) are the main cytokine-producing NK cells (1). In peripheral blood (PB), up to 90% of NK cells are CD56^dim^ NK cells while most NK cells are CD56^bright^ NK cells in lymph nodes.

Natural killer cell functions are regulated by signals delivered through activating and inhibitory receptors. As opposite to T cells, NK cells are “ready to go” and can eliminate target cells without prior stimulation. However, stimulation of NK cells by cytokines leads to NK cell activation and enhanced functions, in particular enhanced cytolytic activity and proliferation. NK cells have long been considered potential candidates for cancer immunotherapy and their versatility makes them attractive cells to explore. Phase I clinical trials showed autologous NK cell therapies to be feasible and safe without adverse effects in patients with breast cancer or non-Hodgkin’s lymphoma; however, these therapies had no or little impact on relapse rates (2). The potential impact of NK cell alloreactivity in hematopoietic stem cell transplantation (HSCT) was suggested by Valiante and Parham (3). The first evidence that allogeneic NK cells could exert strong anti-leukemic activity and impact on the outcome of haploidentical transplantation stems from the study of Ruggeri et al. (4) who reported NK cell alloreactivity against leukemic cells while reducing the risk of graft-versus-host disease (GvHD) in the context of human leukocyte antigen (HLA) mismatch settings. Other trials have showed that allogeneic NK cells alone can target different types of cancers such as acute myeloid leukemia (AML), melanoma, renal cell carcinoma, Hodgkin lymphoma (5), breast and ovarian cancer (6), or refractory lymphoma (7). The same group has shown the importance of NK cell expansion *in vivo*, which can be accomplished by infusion of interleukin (IL)-2. However, regulatory T cells were also found to compete for this cytokine and beneficial effects on NK cell expansion were observed when regulatory T cells could be depleted (8). Interestingly, other studies also have indicated that NK cell therapy could also be of interest to treat glioma (9) or neuroblastoma (10).

Umbilical cord blood (UCB) has become an established source of hematopoietic stem cells (11) for transplantation. Advantages for the use of UCB include low risk of viral transmission from donor to recipient, rapid availability of UCB units serving as an immediate “off-the-shelf” product, less stringent requirements for HLA matching, and lower risk of GvHD. However, UCB contains between 10- and 100-fold fewer nucleated cells than other sources of HSC, limiting how many cells of interest can be retrieved from one UCB unit. Interestingly, NK cells are the first lymphocytes to recover after HSCT including after umbilical cord blood transplantation (UCBT) (12). In addition, NK cells are key effectors of the graft-versus-leukemia (GvL) effect. Especially after UCBT, as T cell immune reconstitution is delayed and there is no increased incidence of relapse, it is likely that NK cells are actually the main effectors of the GvL effect in the first year post-UCBT. However, UCB also contains different types of immune cells including NK cells and as we learn more about their specific characteristics, we will identify the conditions which might benefit of an UCB NK cell therapy. This review focuses on providing an overview of the characteristics of UCB NK cells compared to NK cells from PB and explain how they could be used as a cell therapy to cancer.

## Characteristics of UCB NK Cells

**Figure 1:**
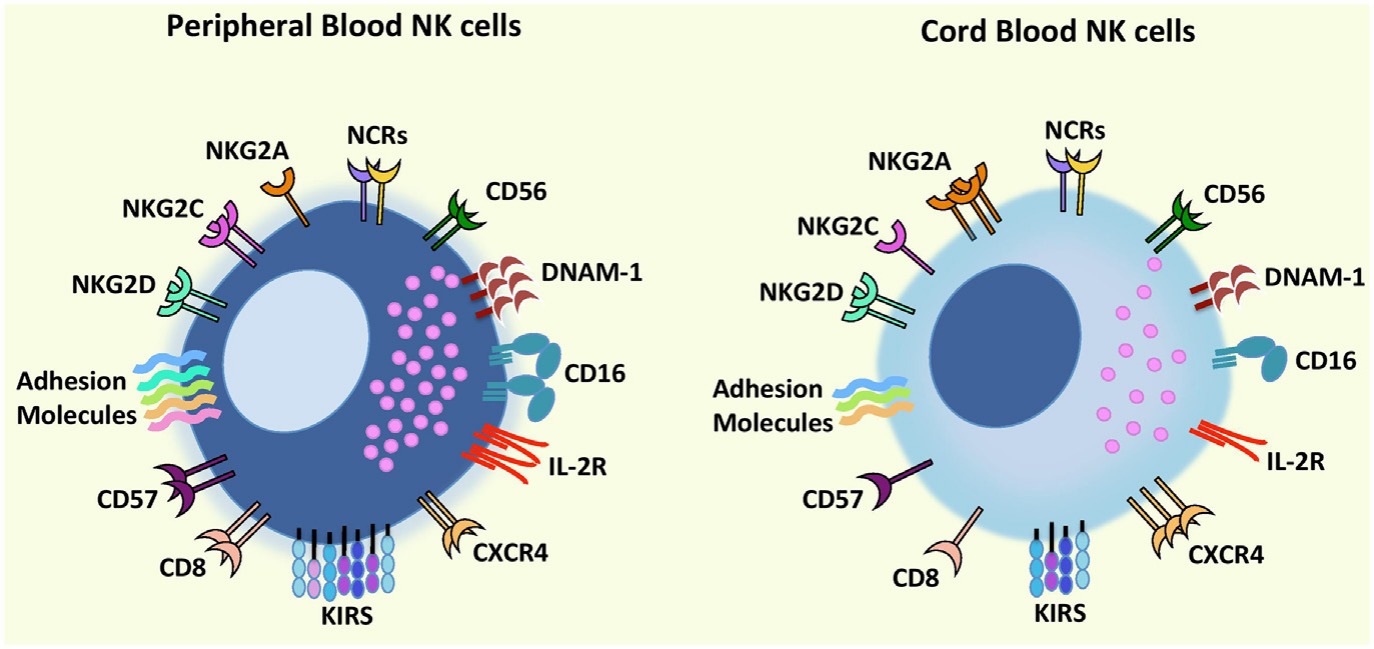
**Comparison of phenotypic characteristics between umbilical cord blood (UCB) natural killer (NK) cells and peripheral blood (PB) NK cells**. In comparison to PB NK cells, UCB NK cells exhibit similar levels of CD56, NCRs (NKp46 and NKp30), and NKG2D but a lower expression of CD16, adhesion molecules (e.g., CD2, CD11a, CD18, CD62L), KIRs, DNAM-1, NKG2C, IL-2R, and CD57 and CD8 (receptors associated with terminal NK cell maturation) together with a higher expression of inhibitory receptor NKG2A indicating that UCB NK cells possess an immature phenotype and reduced cytotoxicity compared to PB NK cells. Further UCB NK cells have a higher expression of the bone marrow homing receptor, CXCR4, compared to PB NK cells proposing that cord blood NK cells may contain a greater potential to home to the bone marrow. Abbreviations: KIRs, killer-cell immunoglobulin-like receptors; NCRs, natural cytotoxicity receptors.

Natural killer cells constitute up to 10% of lymphocytes in PB and up to 30% in UCB (13, 14), and both CD56^dim^ NK cells and CD56^bright^ NK cells can be found in PB and UCB with some groups reporting similar proportions of both subsets or higher frequency of CD56^bright^ NK cells in UCB (14–16). Regarding the phenotype and functions of UCB NK cells, some groups have identified differences when compared to PB NK cells while others found them to be similar to PB NK cells (17) (Figure 1).

### Advantages of UCB-Derived NK Cells

Aside from the higher percentage of NK cells present in UCB, the ability to cryopreserve UCB together with the ease of collecting UCB units offers a unique clinical advantage of making UCB an off-the-shelf source for NK cell immunotherapy. Moreover, a more rapid recovery of NK cells was reported after UCBT than PB HSCT (18, 19). This faster recovery could be explained by the fact that UCB contains different NK cell progenitor populations that have the capacity to differentiate into NK cells and are typically absent in PB (20–22). Further, PB and UCB NK cells produced similar amounts of IFN-γ and TNF-α in response to different stimuli (14, 23) and could proliferate in response to cytokines such as IL-2 or IL-15 (14, 16, 24) despite UCB NK cells exhibiting lower expression of the IL-2 receptor subunits and lower phosphorylation of STAT5 (25). Additionally, UCB NK cells have also been described to have a higher expression of the bone marrow homing receptor, CXCR4, compared to PB NK cells indicating that UCB NK cells may contain a greater potential to home to the bone marrow (14). Finally, IL-15 activated UCB NK cells have been reported to impact positively on UCB HSC engraftment by enhancing their migration and clonogenic capacity, and their engraftment in humanized animal model (26).

### Drawbacks of UCB-Derived NK Cells

The use of cord blood (CB) as a source of NK cells for immunotherapy, however, is also limited as a result of the low numbers and immaturity of CB NK cells. Although, UCB NK cells have been reported to be fully mature and functional (16, 27), some groups found them to have an immature phenotype (14, 28), exhibiting normal levels of degranulation but lower cytotoxicity against K562 cells as compared to PB NK cells (14, 23). This lower activity could be explained by the fact that UCB NK cells have decreased expression of certain adhesion molecules on their surface such as CD2, CD11a, CD18, and CD62L (15, 16), decreased expression of CD16 (15), decreased expression of perforin and granzyme B (14, 23), and lower killer-cell immunoglobulin-like receptors (KIRs) expression together with a higher expression of inhibitory molecules such as NKG2A when compared to PB NK cells indicating an immature phenotype (14, 23). However, activation with cytokines such as IL-2 or IL-15 or the combination of IL-15 with IL-2 or IL-18 was able to restore or enhance their cytotoxicity to the levels observed for PB NK cells (14, 16, 23, 25, 29). Moreover, although the frequencies of NK cells present in UCB is greater than PB (14), low numbers of UCB NK cells are obtained as a result of the limited volume of an UCB unit, which is a major obstacle in obtaining sufficient numbers of NK cells for clinical application. However, different strategies to increase NK cell doses have been developed.

## Expansion of UCB NK Cells

A number of studies have recently explored different platforms to expand UCB NK cells. Increased NK cell numbers can be achieved either by large-scale expansion techniques using artificial antigen-presenting cell (aAPC) or cytokines including IL-2, IL-15, and/or FLT-3 ligand. One such strategy employed to expand purified UCB-derived NK cells on a large scale has been reported using good manufacturing practice (GMP)-grade K562-based aAPCs expressing membrane-bound IL-21 (30). Shah and colleagues have shown that following 14 days of culture in a gas permeable culture system, a 2,389-mean fold expansion of NK cells derived from frozen UCB was achieved. The expanded NK cells presented >95% purity of CD56^+^CD3^−^ NK cells and displayed efficient killing capacity against multiple myeloma *in vitro* and *in vivo*, highlighting the use of aAPCs as an attractive approach to generate large numbers of functionally competent UCB NK cells. A further strategy to evaluate the potential use of expanded NK cells was reported by using aAPCs in the form of genetically modified K562 cells expressing membrane-bound IL-15 and 41BBL (31). The aAPCs were cultured with CB mononuclear cells for 7 days, which led to the generation of expanded UCB NK cells that displayed increased expression of NK cell activating receptors, increased perforin and granzyme expression, and increased cytotoxicity against B-cell non-Hodgkin lymphoma *in vitro* and *in vivo*. The study merits the use of expanded NK cells for adoptive cellular therapy specifically to target relapse or refractory disease after UCBT. Finally, the use of irradiated Epstein–Barr virus-transformed lymphoblastoid cell lines and IL-2 was also recently reported to generate large numbers of CD56^+^ NK cells derived frozen UCB (32). The generated NK cells exhibited higher levels of cytotoxicity against K562 leukemic cells than expanded PB-derived NK cells (32). The unique advantage of this platform is that only 1 ml of the UCB unit is selectively used to generate expanded NK cells for adoptive therapy and the remaining UCB from the same unit can be cryopreserved and used for future transplantation. It would be interesting to assess whether the use of the same UCB for early NK cell adoptive therapy and transplantation can help to prevent relapse and augment GvL post-UCBT.

## Differentiation of NK Cells from UCB CD34^+^ Cells

Natural killer cells can be directly isolated from PB and UCB but an alternative to these cell sources is the differentiation of NK cells from HSC as a way to generate high numbers of cells (33). NK cells can be differentiated from CD34^+^ cells from the bone marrow, from embryonic stem cells, mobilized PB, or UCB CD34^+^ cells. The expansion of NK cells derived from both fresh and frozen UCB CD34^+^ cells using a cocktail of cytokines in a culture system has also been described as an efficient system to generate large numbers of NK cells. We and others have reported the characteristics of NK cells produced *in vitro* from UCB CD34^+^ cells (34–36). These cells are mostly similar to PB NK cells with the exception that they express low levels of inhibitory receptors. However, NK cells produced in such a way have been shown to be functional, able to kill leukemic cell lines and patient cells *in vitro* and *in vivo* and produce cytokines in response to diverse stimuli (34, 36–38). Interestingly, NK cells produced *in vitro* have been shown to expand to high numbers while preserving their phenotype and functions after cryopreservation (39). Thus, frozen UCB CD34^+^ cells were found to be the best source of NK cells when compared to fresh UCB-derived CD34^+^ cells and frozen PB CD34^+^ cells and could therefore be a readily available off-the-shelf product for NK cell immunotherapy.

### NK Cells Alloreactivity in UCBT Setting

Umbilical cord blood NK cells express both inhibitory and activating receptors, which are highly important in mediating self-tolerance or NK cell activity (40). Inhibitory receptors are part of the immunoglobulin superfamily including the KIRs, the immunoglobulin-like transcripts, and C-type lectin receptors CD94/NKG2A. Inhibitory receptors recognize the classical MHC class I molecules on target cells and inhibit NK cell lysis (41). Most KIRs are inhibitory receptors but a limited number of KIRs also function as activating receptors; however, the function and ligands of the later are less well understood. Since KIR genes are not on the same chromosome as HLA, these genes are inherited independently. This allows for donor and recipient HLA-matched UCBT and mismatching between KIRs and their ligands, maintaining the appropriate matching required for HSCT but providing NK cell alloreactivity, which triggers NK cell activation leading to tumor cell lysis (42). This phenomenon of NK cell alloreactivity was proposed as beneficial in reducing relapse after HSCT; however, variable results have been reported from different studies (4, 43–47). In UCBT setting, only few studies have evaluated the outcome of UCBT using mismatched KIR and its ligands (48–51) with only some of them reporting beneficial results (52, 53). KIR haplotype has also been shown to influence the outcome of HSCT. In this context, the higher the number of activating KIR a donor has the higher NK cell alloreactivity might be. Some studies have reported the beneficial effect of the donor B haplotype that contains more activating gene than a A haplotype on HSCT outcome in particular showing a lower incidence of relapse for patients with AML or lower GvHD incidence depending on the study considered (47, 54–56). Whether KIR haplotype can also influence UCBT outcome needs to be investigated.

Finally, NK cell licensing (57), arming/disarming (58), or education (59) is another factor to be considered. NK cells can express one or more inhibitory receptors recognizing HLA molecules. The process by which NK cells become functional and tolerant to self-HLA can be referred to as NK cell licensing and is defined by the fact that to be functional NK cells must express inhibitory receptors recognizing self-HLA. This concept has been well studied in mice and there are now also evidence in humans (59, 60). However, it has been reported that unlicensed NK cells are able to mount an immune response against cytomegalovirus in mice (61) and can kill neuroblastoma cells in humans (10). Therefore, moving forward it will be essential to gain a better understanding of the impact of NK cell licensing on their functions especially in the context of HSCT including UCBT.

## Current Clinical Studies Involving UCB NK Cells

Natural killer cells can be isolated from UCB based on CD56 purification methods. One step isolation method can be used in UCB as opposite to PB where two steps are needed in order to eliminate NKT cells. This is not necessary when considering UCB as it contains a very low percentage of that cell subset. In addition, UCB has the advantage of being readily available as UCB is cryopreserved and can be obtained from accredited UCB banks. Therefore, a NK cell product derived from UCB has the potential to be off-the-shelf. Another advantage of UCB is that HLA is less stringent, although it is not clear what level of matching will be necessary to develop a third party NK cell product from UCB. However, because of the limited volume of blood collected from the umbilical cord there are only a limited number of NK cells that can be isolated from UCB. In addition, as they are immature and have lower functionality as compared to PB NK cells; taking UCB NK cells to the clinics will require a prior activation/expansion step. Several clinical trials are currently ongoing to evaluate the safety and feasibility of UCB NK cells as an “off the shelf product” in transplant and non-transplant settings (Table 1). GMP grade expansion methods for UCB NK cells are currently available as previously described. Notably, only a handful of clinical trials are currently ongoing and recruiting patients using the latest method to expand UCB NK cells to reach the cell dose required. Two clinical phase I studies aim to use expanded UCB NK cells for the treatment of patients with chronic lymphocytic leukemia (NCT01619761, NCT02280525), while another aims to evaluate NK cell therapy in the context of autologous HSCT for patients with myeloma (NCT01729091).

**Table 1:** **UCB NK cells currently in the clinic**.

Clinical trial identifier	Diseases	Trial phase	Type of transplant	Conditioning	Method of expansion	Sponsor
NCT01619761	ALL, AML, CLL, CML, HL, MDS, MM, NHL, SLL	I	Double umbilical cord blood transplantation	Fludarabin, melphalan, lenalidomide ± rituximab	*Ex vivo* expansion of NK cells from 20% UCB unit fraction	MD Anderson Cancer Center
NCT02280525	CLL, ALL, AML, CML, NHL, HL	I	Non-HSCT	Fludarabin, cyclophosphamide, lenalidomide, and rituximab	*Ex vivo* expansion of NK cells from thawed from UCB unit	MD Anderson Cancer Center
NCT01729091	MM	I//II	Autologous	Melphalan, lenalidomide	*Ex vivo* expansion of NK cells from thawed from UCB unit	MD Anderson Cancer Center
EudraCT number 2010-018988-41	AML	I	Non-HSCT	Fludarabin, cyclophosphamide	NK cells generated *in vitro* from UCB progenitor cells	Radboud Medical Centre, Nijmegen, Netherlands

*ALL, acute lymphoblastic leukemia; AML, acute myeloid leukemia; CB, cord blood; CLL, chronic lymphoblastic leukemia; CML, chronic myeloid leukemia; HL, Hodgkin lymphoma; HSCT, hematopoietic stem cell transplantation; MDS, myelodysplastic syndromes; MM, multiple myeloma; NHL, non-Hodgkin lymphoma; NK, natural killer; SLL, small lymphocytic lymphoma; UCB, umbilical cord blood*.

Only a few groups have focused on developing cell therapy approaches based on the differentiation of NK cells from HSC *in vitro*. However, NK cells produced *in vitro* have been shown to be safe and their use feasible when considered in the context of allogeneic HSCT (62). In addition, another trial, oNKord^®^, is currently ongoing testing the use of NK cells produced *in vitro* from UCB CD34^+^ cells in patients with AML (EudraCT number 2010-018988-41).

## Concluding Remarks

Immunotherapy is a promising treatment for different types of cancer allowing the possibility of personalized medicine for each cancer patient. UCB provides distinct advantages and is an increasingly attractive source for HSCT and cellular therapy. Despite low NK cell numbers within a single UCB unit and their immature phenotype, strategies to expand UCB NK cells using aAPCs or cytokines and feeder cells are paving the way for NK cell adoptive immunotherapy. NK cells have shown great potential in eliminating different types of cancer cells *in vitro* and in animal models. A few clinical trials are currently underway to evaluate the safety and feasibility of using UCB NK cells as an “off the shelf” product for the prevention of relapse. The results from these studies will help in understanding how to maximize the beneficial potential of UCB NK cells for the treatment of hematological malignancies and solid tumors.

## Author Contributions

All the authors contributed to writing and reviewing the manuscript.

## Conflict of Interest Statement

The authors declare that the research was conducted in the absence of any commercial or financial relationships that could be construed as a potential conflict of interest.
